# Synthesis of multicolor-emitting nitrogen–sulfur co-doped carbon dots and their photochemical studies for sensing applications†

**DOI:** 10.1039/d2ra03073j

**Published:** 2022-07-11

**Authors:** Manunya Tepakidareekul, Taro Uematsu, Susumu Kuwabata

**Affiliations:** Department of Applied Chemistry, Graduate School of Engineering, Osaka University 2-1 Yamada-oka Suita 565-0871 Japan t-uematsu@chem.eng.osaka-u.ac.jp kuwabata@chem.eng.osaka-u.ac.jp; Innovative Catalysis Science Division, Institute for Open and Transdisciplinary Research Initiatives (ICS-OTRI), Osaka University Suita 565-0871 Japan

## Abstract

Photoluminescent carbon dots (CDs) possess several advantages, which include high stability and a non-toxicity that are essential in different applications such as catalysis, drug delivery, and sensors. The presence of heteroatoms modifies their physicochemical characteristics. In this work, a combination of CDs is manufactured utilizing a solvothermal technique using citric acid and thiourea. After separating each section using column chromatography, green and yellow CDs with average diameters of 8.3 and 7.0 nm, respectively, are generated. Next, optical and structural characterizations indicated that the variation in the emission color was caused by differences in surface functional groups rather than particle size. The photoelectrochemical properties are explored by including quinone derivatives and metal ions, which are quenchers for the CDs. The photoluminescence quenching results showed the presence of anionic functional groups on the surface of the CDs. Furthermore, these functional groups interacted strongly with particular types of metal ions, indicating that they may be employed as metal ion sensors.

## Abstract 2

Luminescent carbon dots (CDs) have been synthesized by a solvothermal approach and their photochemical properties are evaluated. Responses to specific metal ions are observed, and the relationship with surface functional groups is discussed.

## Graphical abstract

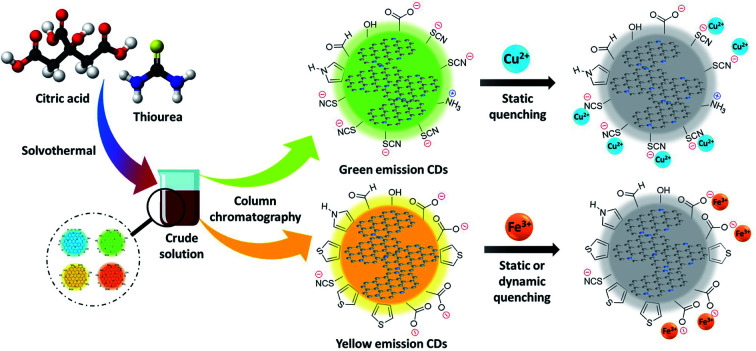

## Introduction

Carbon dots (CDs), a new type of fluorescent nanomaterials, have garnered plenty of interest over the last decade because of their distinctive properties such as emission tunability, high fluorescence quantum yield, chemical inertness, and biocompatibility. CDs are characterized as tiny carbon compounds with a size of less than 10 nm that are largely made up of sp^2^ and sp^3^ carbons with a little quantity of heteroatoms. To alter emission qualities, these heteroatoms provide energy structure modifications.^1–3^ Although numerous approaches to prepare CDs have been devised, the solvothermal method is considered to be one of the most simple processes. When a combination of carbon sources, including phenylenediamine, citric acid, urea, hydroquinone, ethylenediamine, and even lemon juice, is reacted in common solvents such as ethanol, DMF, acetone, and formamide, CDs with varied emission colors are generated owing to heteroatom variation.^4–6^ Basically, photon absorption and emission occur in the sp^2^-conjugated sections of the carbon centers, and the photoluminescence (PL) property depends on the size.^7–9^ Doping heteroatoms changes the electron orbitals of both sp^2^ moieties and surface functional groups, resulting in changes in the PL characteristics.^8,10,11^ For example, researchers observed that doping sulfur atoms together with nitrogen enhances the PL intensity.^12–14^ As a result, nitrogen–sulfur co-doped CDs have received extensive research and are used in a variety of applications such as bioimaging,^15–17^ lighting,^18,19^ drug delivery,^20,21^ and chemosensors.^14,22–24^

Heavy metal ions are known for high toxicity, and they are often accumulated in the human body *via* inhalation and food consumption.^25^ Therefore, the detection of trace metal ions in the environment is important for preserving biological systems. Upon construction of detection systems, not only high sensitivity but selectivity toward particular metal ions is required, and several sensing systems using different detection methods, such as electrochemistry^26,27^ and optics,^28–31^ have been proposed. Among the optical sensors, fluorescence detection using CDs as fluorophores has been extensively adopted due to high selectivity, sensitivity, and fast response, as well as technical simplicity.^32,33^ Many of them use PL intensity variations due to the approach of metal ions; therefore, interactions between CDs and metal ions essentially determine the sensing performances. Typically, PL quenching depends on specific chemical features of the CDs, such as surface functional groups and the redox potential of energy levels. Hence, examinations of PL intensity variations due to the addition of various quenchers will offer extensive information about the CDs. Sun *et al.* created nitrogen–sulfur co-doped CDs from l-cysteine and utilized them to detect Co^2+^ upon the addition of Co^2+^, and the complexation between the surface functional groups of the CDs with Co^2+^ occurred and exhibited the quenching of the PL.^34^ Also, Sun *et al.* created nitrogen–sulfur co-doped CDs from heparin sodium and utilized them for sensing Fe^3+^.^29^ As listed in Table 1, the majority of these experiments have been conducted with blue-emission CDs, and only a few cases have been tested with different hues. Because the emission color and surface qualities are so closely coupled, if the sensitivity and selectivity against various chemicals vary depending on the emission color, which would be useful for both understanding CDs and designing better chemosensors, is worth investigation.

**Table 1:** Synthetic conditions, optical, and detection characteristics of nitrogen–sulfur co-doped CDs-based fluorescence sensors

Precursor	Synthesis method	Excitation (nm)	Emission (nm)	Emission color	Analyte	LOD (μM)	References
l-cysteine	Hydrothermal	340	440	Blue	Co^2+^	0.0026	34
Sodium citrate and sodium thiosulfate	Hydrothermal	350	440	Blue	Fe^3+^	0.1	35
Heparin sodium	Hydrothermal	325	390	Blue	Fe^3+^	—	29
Citric acid and taurine	Hydrothermal	345	443	Blue	Hg^2+^	0.05	36
Garlic	Hydrothermal	340	428	Blue	Fe^3+^	0.00022	24
Citric acid and thiourea	Microwave-assisted	358	436	Blue	Hg^2+^	1.78	30
Citric acid and thiourea	Microwave-assisted	360	443	Blue	Fe^3+^	0.16	37
Citric acid and thiourea	Microwave-assisted	416	523	Greenish-blue	Hg^2+^	0.072	31
Citric acid and thiourea	Solid state carbonization	360	436	Blue	Ag^+^	12.9	38
					Hg^2+^	9.4	
Citric acid and thiourea	Hydrothermal	340	445	Blue	Hg^2+^	0.0014	39
Citric acid and thiourea	Solvothermal in DMF	560	594	Red	Fe^3+^	0.0097	40
Citric acid and thiourea	Solvothermal in DMF	425	540	Green	Cu^2+^	10	This work
		450	567	Yellow	Fe^3+^	113	This work

This study aims to provide one-pot synthesis of the CDs with green and yellow emissions by solvothermal synthesis using citric acid and thiourea in the DMF solution. During the process, the precursors undergo hydrolysis and carbonization to form numerous types of carbon dots, which are isolated using column chromatography. To characterize the CDs, structural and optical approaches are used. In addition, PL properties and sensing mechanism of these CDs are investigated by PL quenching upon the addition of electrochemically active species in various situations.

## Experimental section

### Materials

Citric acid was supplied by TCI. Thiourea, DMF, 2-amino-3-chloro-1,4-napthoquinone, disodium anthraquinone-1,5-disulfonate, silver nitrate (AgNO_3_), cadmium nitrate (Cd[NO_3_]_2_·4H_2_O), cobalt(ii) chloride (CoCl_2_·6H_2_O), nickel(ii) nitrate (Ni[NO_3_]_2_·6H_2_O), and lead(ii) nitrate (Pb[NO_3_]_2_), and zinc nitrate (Zn[NO_3_]_2_·6H_2_O) were purchased from Fujifilm Wako Chemicals. Copper(ii) perchlorate (Cu[ClO_4_]_2_·6H_2_O), iron(iii) chloride (FeCl_3_·6H_2_O), and manganese(ii) chloride (MnCl_2_·4H_2_O) were purchased from Kishida chemicals. Next, iron(ii) chloride (FeCl_2_·4H_2_O) was supplied by Kanto chemicals. Dichloromethane (CH_2_Cl_2_), methanol (MeOH), ethyl acetate (EtOAc), and diethyl ether (Et_2_O) were purchased from Fujifilm Wako Chemicals. Water used in the present study was purified by a Milli-Q Integral 3 (Merck) with a resistivity of greater than 18.2 MΩ cm.

### Instruments

The morphologies of the prepared CDs were studied by a Hitachi H-7650 transmission electron microscope (TEM). The average particle size was determined by measuring at least 200 particles in TEM images. The KBr pellet technique was used to record Fourier transform infrared (FT-IR) spectra on a PerkinElmer Spectrum 100 FT-IR Spectrometer. X-ray diffraction (XRD) patterns were captured using a powder X-ray analysis system (Rigaku, SmartLab) equipped with a parallel-beam/parallel-slit analyzer. X-ray photoelectron spectra (XPS) were collected with an X-ray photoelectron spectrometer (Shimadzu, KRATOS AXIS-165x) fitted with an aluminum target. A Jasco FP-8600 spectrofluorometer was used to capture the PL spectra. A Jasco V-670 spectrophotometer was used to record the ultraviolet-visible (UV-vis) absorption spectra. The PL lifetimes were calculated from time-resolved spectra obtained using a Hamamatsu Quantaurus-Tau C11367-22. The absolute PL quantum yield (QY) was evaluated using a PMA-12 (Hamamatsu) equipped with an integrating sphere.

### Synthesis of CDs

The CDs were synthesized by mixing 4.2 g (0.02 mol) of citric acid and 7.6 g (0.10 mol) of thiourea with 100 mL of DMF. Before transferring the mixed solution to a Teflon-lined autoclave, it was agitated for 15 minutes. After heating the solution at 160 °C for 24 h, it was allowed to cool to an ambient temperature. A 0.45 μm polytetrafluoroethylene syringe filter was used to filter out large particles from the crude solution. Following that, a 1 : 5 Et_2_O : EtOAc mixture was added in excess to precipitate big particles, which were removed by centrifugation. The supernatant was collected and purified using column chromatography with gradient elution method using CH_2_Cl_2_ and MeOH. Next, the CDs were divided into four portions that revealed four various colors of blue, green, yellow, and orange, which were labeled as b-CDs, g-CDs, y-CDs, and o-CDs, before being distributed in the water.

### PL quenching by quinone derivatives

Aqueous solutions of the g-CDs and y-CDs were separately prepared at the concentration of 0.4 mg mL^−1^. 2-Amino-3-chloro-1,4-napthoquinone and disodium anthraquinone-1,5-disulfonate were separately dissolved in water at the concentration of 1.25 mM, which were the stock solutions of the quenchers. Next, PL spectra of the CDs were determined in the absence and presence of the quenchers.

### Metal selectivity tests

The 0.1 M aqueous solutions of AgNO_3_, Cd(NO_3_)_2_, CoCl_2_, Cu(ClO_4_)_2_, FeCl_3_, MnCl_2_, Ni(NO_3_)_2_, Pb(NO_3_)_2_, and Zn(NO_3_)_2_ were prepared separately as stock solutions. These metal-ion solutions were mixed with the solutions of the CDs (0.4 mg mL^−1^) with the concentration of up to 5 mM, and the PL intensities were measured using the spectrofluorometer. For specific combinations, PL decay curves were measured and the variations in lifetimes and PL intensity were compared. The PL quenching tests were also performed in different temperatures to investigate the interaction between the CDs and quenchers.

## Result and discussion

### Characterization of CDs

Citric acid and thiourea, which are precursors for the solvothermal synthesis of the CDs, undergo dehydration and carbonization at higher temperatures (>160 °C) to form photoluminescent CDs consisting of multiple aromatic rings. The as-prepared solution revealed white PL under UV irradiation, and its PL peak wavelength varies depending on the excitation wavelength (Fig. S1†). These results showed that numerous different types of CDs were produced during heating and column chromatography was conducted to separate the crude product into four sections exhibiting blue, green, yellow, and orange color emissions (Fig. S2†). Because the quantities of b-CDs and o-CDs were low, a quantifiable amount could not be obtained after an extra purifying step. Therefore, the g-CDs that exhibited the highest PL QY (Table S1,† in acetonitrile), and the y-CDs that were the major product under the present synthetic conditions, were chosen for further analysis.

**Fig. 1:**
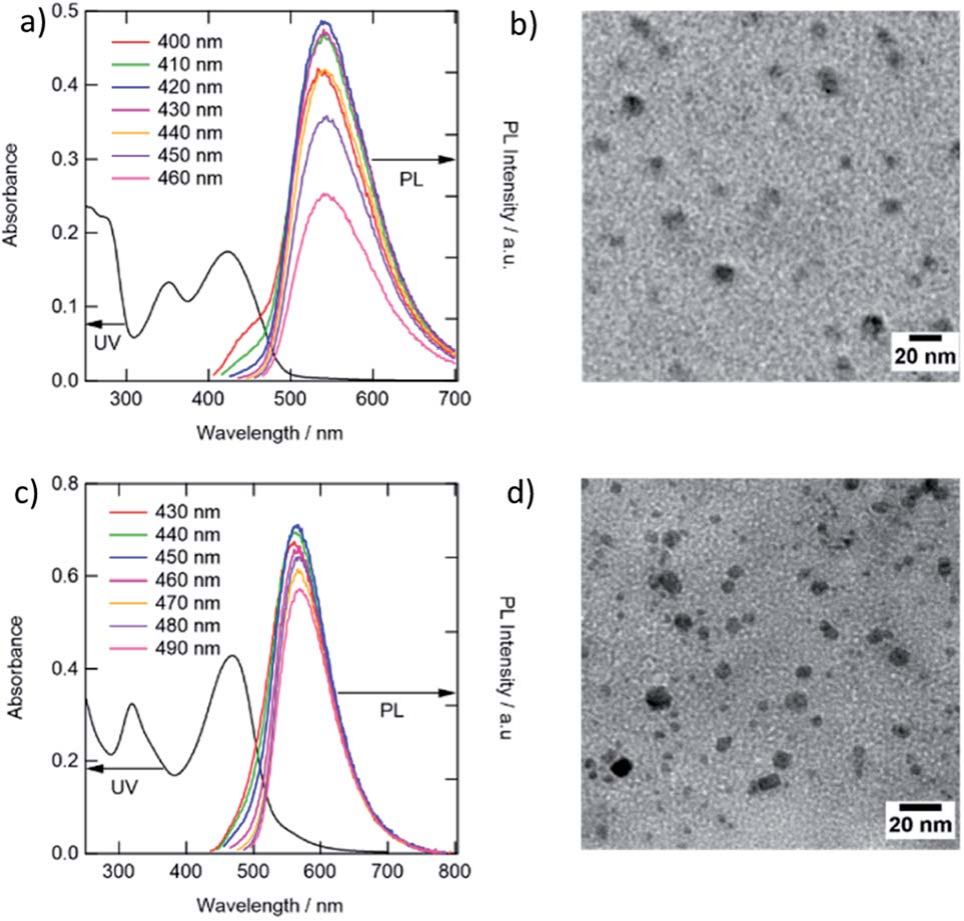
UV-vis and PL spectra, and TEM images of the g-CDs (a, b) and y-CDs (c, d). The PL spectra were recorded as a function of different excitation wavelengths in aqueous solution (pH 7.0).

The UV-vis absorption spectra of the g-CDs and y-CDs reveal distinct absorption profiles (Fig. 1a and c). The absorption bands corresponding to the π–π* transition of the graphitic sp^2^ carbon and the n–π* transition of the conjugated C=O appeared in the UV region. Furthermore, typical absorption bands centered at 425 nm and 470 nm for the g- and y-CDs were observed. These bands could be attributed to the n–π* transition of the conjugated C=N and C=S bonds, which is usually present in the nitrogen–sulfur co-doped CDs prepared by a solvothermal process using citric acid and thiourea.^13,40,41^ Hence, CDs are thought to have several emission states in a single particle, resulting in the excitation wavelength dependency of the emission spectra illustrated in Fig. S3.† When the excitation wavelength is tuned to UV, the chromophore with C=O bonds is stimulated, producing blue color emission (400–450 nm).^7–11,42^ Conversely, when the same CDs were stimulated around the significant absorption peaks locating in the visible region (400–460 nm for the g-CDs and 430–490 nm for y-CDs), green (540 nm) and yellow (567 nm) emissions were generated, respectively. Positions of these PL peaks were independent of the stimulation wavelength (Fig. 1a and c), and the shapes of PL excitation spectra of each sample (Fig. S4†) were comparable to the absorption spectra in the wavelength region longer than 300 nm (Fig. 1). These findings revealed that the CDs' electronic levels had degraded to some extent. However, they were different from semiconductor quantum dots, which had a band structure and a distinct quantum size effect, which is obvious from the similarity in the particle diameters between the g-CDs (8.3 nm) and y-CDs (7.0 nm) (Fig. 1b and d).

**Fig. 2:**
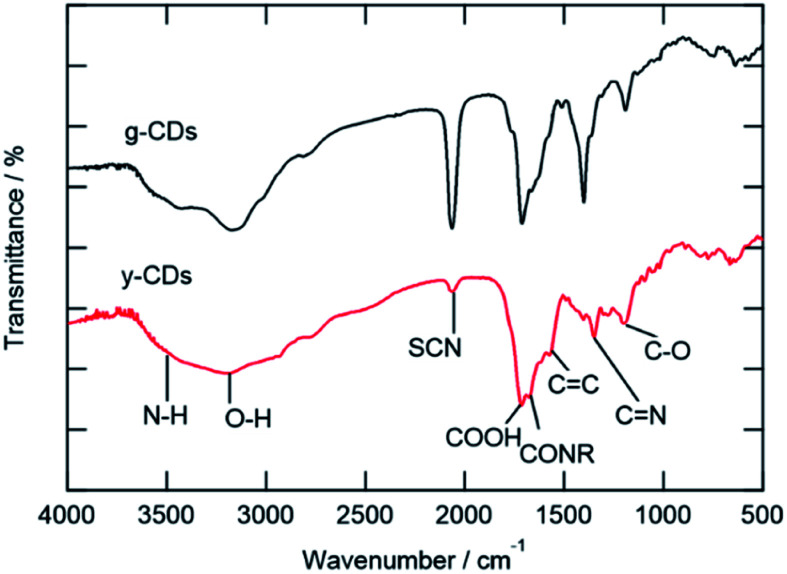
FT-IR spectra of the g-CDs and y-CDs.

**Fig. 3:**
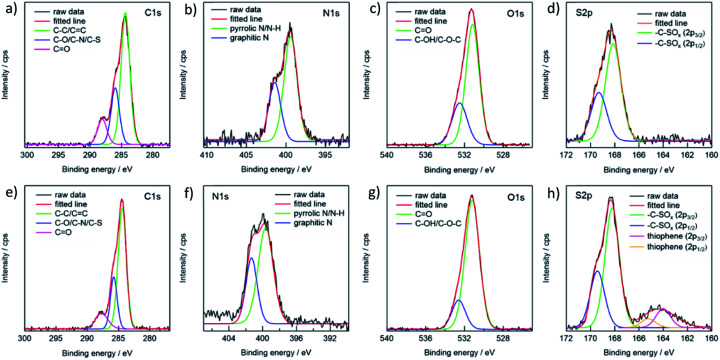
XPS spectra of the g-CDs and y-CDs for C 1s (a, e), N 1s (b, f), O 1s (c, g) and S 2p (d, h) regions.

The FT-IR and XPS spectra are shown in Fig. 2 and 3, respectively. The FT-IR spectra showed that both types of CDs have several kinds of hydrophilic moieties, such as N–H (approximately 3500 cm^−1^), O–H (3194 cm^−1^), COOH (1712 cm^−1^), and CONR (1670 cm^−1^) resulting in good dispersity in aqueous media (Fig. 2). Meanwhile, the stretching vibration of –SCN (2070 cm^−1^), C=C (1575 cm^−1^), and C=N (1403 cm^−1^) groups were observed, confirming the presence of polyaromatic structure formed during the reaction process. A comparison of the two spectra reveals two crucial facts. First, the y-CD's O–H stretching vibration band, which occurred about 3200 cm^−1^, was wider than that of the g-CDs, indicating the presence of multiple kinds of hydroxyl groups on the surface of the y-CDs due to a higher degree of oxidation.^8^ Secondly, the relative intensity of –SCN moiety of the y-CDs was significantly weaker than that for the g-CDs, showing that these groups in the y-CDs were transformed into sulfone or thiophene.

The presence of nitrogen and sulfur was verified by XPS analysis for both the g- and y-CDs. The spectra of the C1s region could be deconvoluted into three peaks, which were assigned to C–C or C=C (284.4 eV), C–O or C–N or C–S (286.0 eV), and C=O (288.1 eV) groups (Fig. 3). The spectra in the N 1s region exhibited two peaks, located at 399.4 and 401.4 eV, and were assigned to pyrrolic N or N–H and graphitic N, respectively. The g-CDs revealed a single kind of sulfur (S 2p), yielding two peaks (168.1 and 169.3 eV) that are assigned to S 2p3/2 and 2p1/2 of –C–SO_*x*_–C– sulfone bridges.^13,40,41,43^ The spectrum for the y-CDs exhibited small peaks at lower energy levels (164.3 eV), which could be assigned to thiophene, consistent with the FT-IR data. As a result, the sulfur content of the y-CDs was greater (4.83%) than that of the g-CDs (3.66%), as indicated in Table 2. As previously reported, these findings suggested that sulfur plays an important role in defining PL characteristics.^12–14^

**Table 2:** Chemical composition determined by XPS

Atomic concentration (%)	C	N	O	S
g-CDs	55.16	10.93	30.26	3.66
y-CDs	55.83	9.86	29.49	4.83

### PL quenching mechanism

One of the possible applications of CDs is a chemosensor, particularly for detecting metal ions that take the merit of a variety of surface functional groups on the CDs. While certain CDs have excellent selectivity against metal ions, the mechanism of PL intensity variations has not been well examined. Typically, PL quenching of CDs by electrochemically active substances can be either dynamic or static. The former (dynamic quenching) is an electron or energy transfer process that occurs when a photoexcited CD collides with a ground state quencher, and the amplitude of the quenching is determined by the interaction of the CDs and the quenchers.^44,45^ In the latter case (static quenching), the quenchers are attached to the ground state CDs, which generate non-luminescent complexes. The static process exhibits a higher magnitude of the quenching (higher sensitivity against the quencher) as a result of the absence of the slow diffusion process. In this case, the magnitude of the quenching is typically determined by the potential gap of the corresponding orbitals (in case of electron transfer) or spectral overlap (in case of Förster energy transfer) of donors and acceptors. For understanding the phenomenon, the correlation between the PL intensity variations and the concentration of the quencher, which is known as the Stern–Volmer-type plot, is frequently used.1
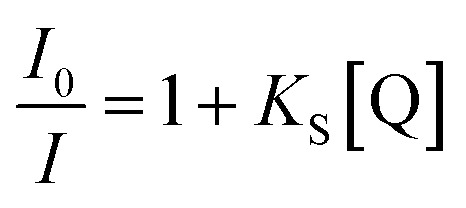
where *I*_0_ and *I* are the PL intensities in the absence and presence of quencher, respectively. *K*_S_ is the quenching constant and [Q] is the concentration of the quencher, which is an analyte in the chemosensing.

**Fig. 4:**
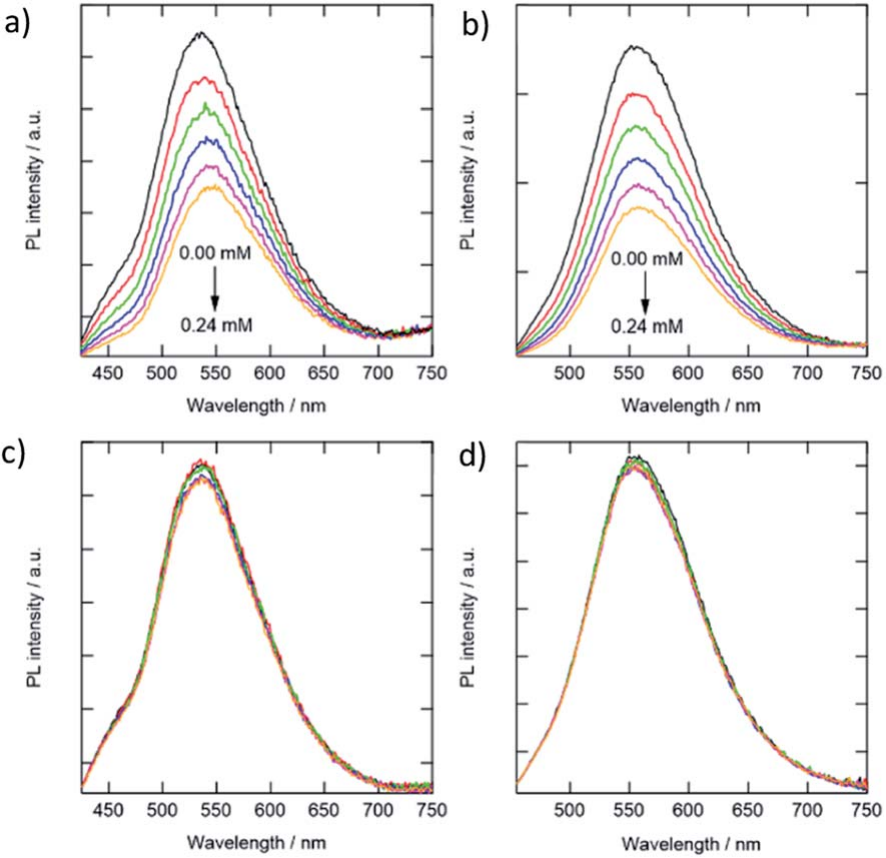
PL intensity variations of the g-CDs and y-CDs in the presence of 2-amino-3-chloro-1,4-naphthoquinone (a, b) and anthraquinone-1,5-disulfonate (c, d) in aqueous solution (pH 7.0).

In the beginning, we examined the effect of electrostatic interactions between the CDs and analytes. Ionic quinone derivatives were utilized to analyze the electrostatic effects and identify the ionic charge of the CDs to avoid the complexation of the analytes with a specific functional group of the CDs. Fig. 4 reveals the PL intensity variations of the CDs (the g- and y-CDs) in the presence of quinone derivatives. The quenching coefficients (*K*_S_) for cationic 2-amino-3-chloro-1,4-naphthoquinone were as high as 5038 and 5224 M^−1^ for the g-CDs and y-CDs, respectively. When anionic anthraquinone-1,5-disulfonate was combined with the g-CDs and y-CDs, no noticeable PL quenching was seen. Because the spectral overlaps between the quencher's optical absorption and the PL of the CDs are low, the occurrence of fluorescence resonance energy transfer is excluded.^45^ Therefore, the photoinduced electron transfer from the CDs to the quenchers as a quenching process seems possible. Furthermore, the cationic and anionic quenchers possess very similar reduction potentials to one another, *i.e.*, −0.170 V for anthraquinone-1,5-disulfonate^46^ and −0.177 V for 2-amino-3-chloro-1,4-naphthoquinone as a function of the standard hydrogen electrode at pH = 7.^47^ These findings pointed to the anionic environment of CDs derived from carboxylate and thiocyanate groups. The magnitudes of quenching by the cationic quencher were between the dynamic (∼10^2^ M^−1^) and static (∼10^5^ M^−1^) processes, showing substantially strong interaction with the cationic quenchers. As a result, the repulsive force created by the overlap of electric double layers becomes greater than that of tiny molecules, resulting in the PL's intactness in the presence of anionic quenchers.

**Fig. 5:**
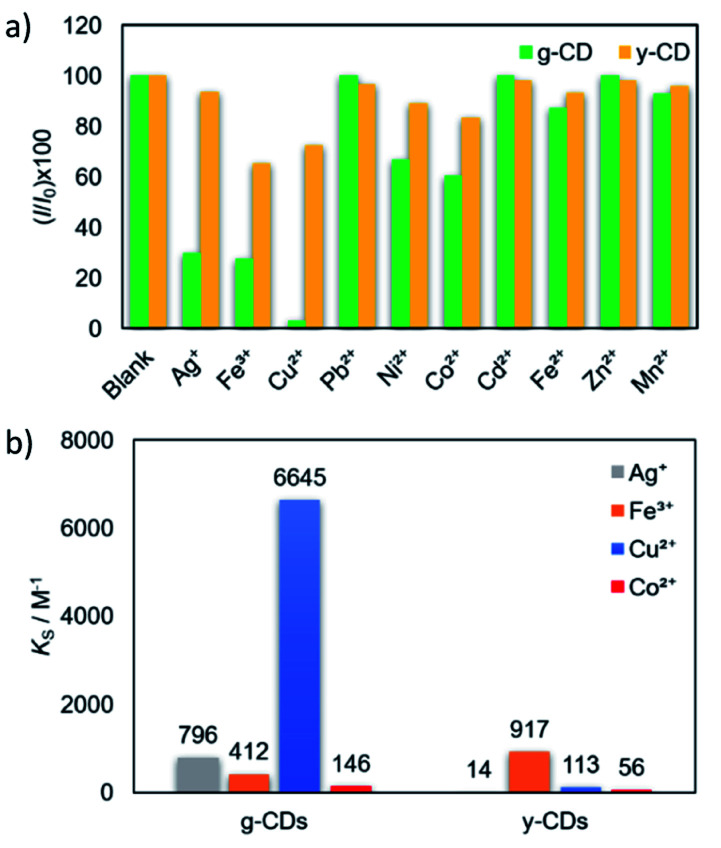
Quenching efficiency of the g-CDs (green bars) and y-CDs (yellow bars) upon the addition of 5 mM various metal ions (a). *K*_S_ values recoded for the g-CDs and y-CDs by the addition of the selected metal ions (b).

The selectivity of the CDs for metal detection was examined by adding various metal ions in 5 mM (Fig. 5a). The magnitudes of quenching significantly varied by metal ions, which were evaluated as a function of (*I*/*I*_0_) × 100. Basically, attractive force is expected between anionic CDs and metal cations. However, several metal ion species (Ag^+^, Co^2+^, Cu^2+^, and Fe^3+^) exhibited high to moderate quenching magnitudes Among these, the PL intensity variations of the g-CDs and y-CDs by the addition of the selected four metal ion species were shown in Fig. S5 and S6,† and the values for *K*_S_ (the slopes of the Stern–Volmer-type plots) were summarized in Fig. 5b. Notably, the g-CDs exhibited the highest *K*_S_ (6645 M^−1^) for Cu^2+^, which was more than 10 times higher than that observed for Co^2+^ (146 M^−1^). In contrast, the y-CDs recorded the highest *K*_S_ by Fe^3+^ (917 M^−1^), and that by Cu^2+^ was very low (113 M^−1^).

**Fig. 6:**
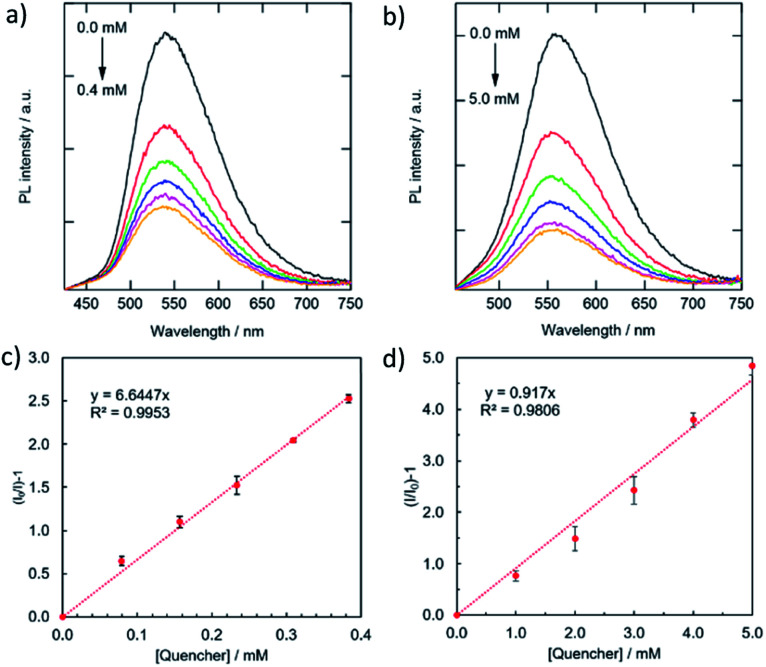
Variations in PL spectra (a, b) and the corresponding Stern–Volmer-type plots of the quenching (c, d) for the combinations of the g-CDs with Cu^2+^ (a, c) and the y-CDs with Fe^3+^ (c, d).

For further understanding of the relationships between the magnitude of quenching and the types of metals, the *K*_S_ values were compared with the reduction potential of the metal ion species. If quenching occurs by electron transfer from photoexcited CDs to the lowest unoccupied molecular orbital (LUMO) of adsorption quenchers, the amount of quenching is predicted to rise with a positive shift in metal cation reduction potential.^48,49^ However, the g-CDs indicated irregular variations in the magnitude of PL quenching, *i.e.*, the highest *K*_S_ (6645 M^−1^) was obtained by Cu^2+^ (0.34 V *vs.* SHE), whereas that by Ag^+^ (0.80 V) and Fe^3+^ (0.77 V) were 10 times lower (796 M^−1^ and 412 M^−1^, respectively). Even when the intensity of the electrostatic interactions is considered, *i.e.*, Fe^3+^ ions should have the highest attractive force with negatively charged g-CDs of the three. These results indicated that the high selectivity of the g-CDs toward the detection of Cu^2+^ is due to the unique structure of the g-CDs, which is the presence of thiocyanates groups on their surface, which is known to improve the adsorption of specific metal cations like Cu^2+^.^40,50^ These arguments favored the development of non-luminescent complexes as the mechanism of quenching rather than electron transfer. The limit of detection was calculated based on the standard deviation of repeated blank measurements and the slope of calibration curve,^51^ and it was found to be 10 μM. This sensing range is good to determine excess Cu^2+^ content in drinking water according to the standard by world health organization.^52^ In the case of the y-CDs, the highest *K*_S_ was recorded by Fe^3+^ (0.77 V), and it decreased by Cu^2+^ (0.34 V) and Co^2+^ (−0.277 V) in this order. However, among these metal ions, Ag^+^, which has the highest reduction potential (0.80 V), did not exhibit PL quenching. These findings suggested that the magnitudes of quenching had no bearing on the reduction potentials. The limit of detection was calculated to be 0.11 mM; 10 times higher than that for the g-CDs against Cu^2+^ due to weaker interaction with the carboxyl groups.

**Fig. 7:**
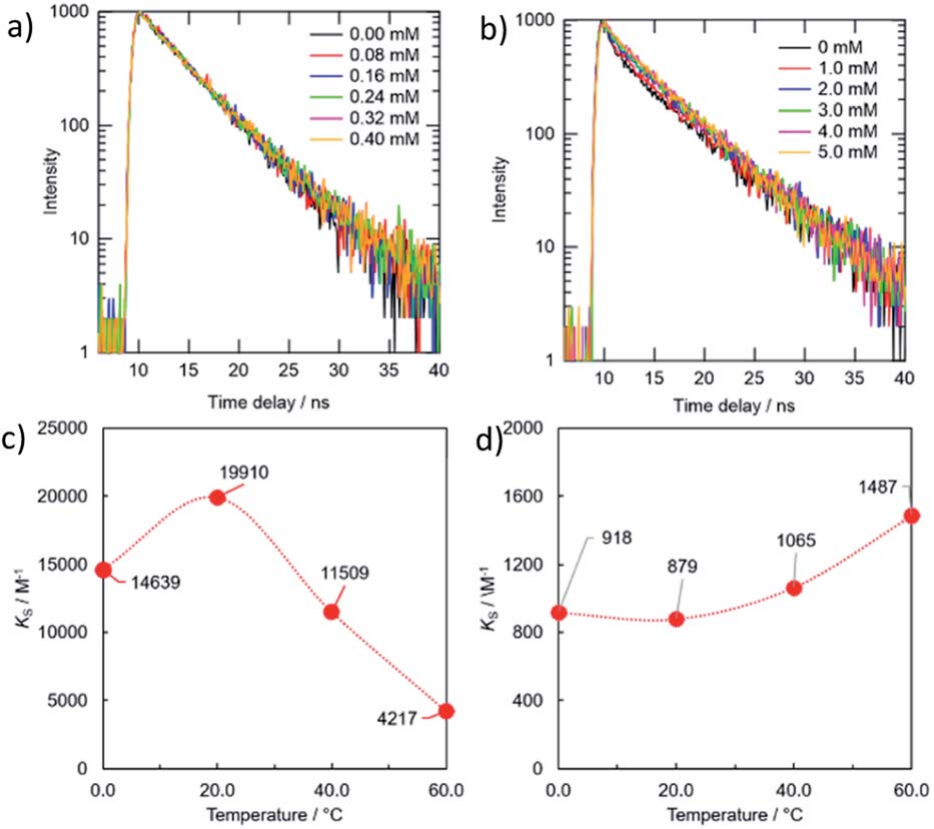
PL decay curves (a, b) and variations in *K*_S_ values (c, d) under different temperatures for the combinations of the g-CDs with Cu^2+^ (a, c) and the y-CDs with Fe^3+^ (c, d).

**Scheme 1:**
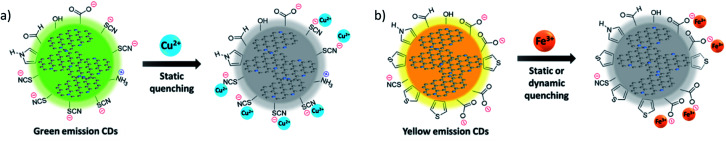
Schematic illustrations showing the differences in sensing behavior between (a) g-CDs and (b) y-CDs that have different surface functional groups. The quenching mostly occurs *via* complexation with metals (static quenching) and partly *via* electron transfer (dynamic quenching).

Fig. 6 and 7 showed the PL spectra and the decay curves for the g-CDs and y-CDs in the presence of Cu^2+^ and Fe^3+^, respectively. As previously stated, the PL intensities of the CDs declined as metal concentration increased, resulting in the straight line in the Stern–Volmer-type graphs (Fig. 6c and d). Conversely, the PL decay curves maintained unchanged (Fig. 7a and b), supporting the formation of non-luminescent complexes rather than electron transfer as expected from the lack of relevance to the reduction potential of the quenchers. The quenching of the g-CDs was discovered to have an evident contribution from static quenching due to the drop in *K*_S_ values with increasing temperature (Fig. 7c). Conversely, the small increase of the *K*_S_ value, which was discovered for the quenching of the y-CDs at a higher temperature, indicated the presence of the dynamic quenching (Fig. 7d). In any event, the quenching of CDs by metal ions was regulated by metal ion binding and the creation of non-luminescent complexes as shown in Scheme 1.

## Conclusions

A solvothermal technique utilizing citric acid and thiourea dissolved in DMF was used to effectively produce nitrogen–sulfur co-doped CDs. By using column chromatography *via* a gradient elution approach, crude compounds were purified and separated, and CDs with green and yellow emissions were obtained and studied. TEM observation showed that the two types of CDs were similar in size; 8.3 ± 1 nm and 7.1 ± 2 nm for the g-CDs and y-CDs, respectively. These findings suggested that changes in emission color were not caused by the quantum confinement effect. The XRD, IR, and XPS spectra indicated that the CDs exhibited comparable functional groups, such as carboxylic, amino, thiocyanate, and polyaromatic carbons, but the ratios of these moieties varied depending on the emission colors. The PL quenching tests performed by the addition of ionic electron acceptors (quinone derivatives) demonstrated that the two types of carbon dots possessed anionic functional groups on their surface. They were susceptible to metal ions in different ways because of the differences in surface functional groups. Cu^2+^ considerably quenched the g-CDs, while Fe^3+^ was the most effective in quenching the y-CDs. A detailed analyses of the quenching by metal ions showed that it was caused by the formation of non-luminescent complexes rather than by the electron transfer between the CDs and metal ions.

## Conflicts of interest

There are no conflicts to declare.

## Supplementary Material

RA-012-D2RA03073J-s001
