# Supplementary material for: Synthesis of multicolor-emitting nitrogen–sulfur co-doped carbon dots and their photochemical studies for sensing applications
Source: RSC Adv. 2022 Jul 11;12(31):20054–61. doi: 10.1039/d2ra03073j (PMC9272668; doi:10.1039/d2ra03073j)
Supplement: RA-012-D2RA03073J-s001 [file RA-012-D2RA03073J-s001.pdf]

Electronic Supplementary Material (ESI) for RSC Advances.
This journal is © The Royal Society of Chemistry 2022

Supplementary information for

Synthesis of multicolor-emitting nitrogen–sulfur co-doped carbon dots and their photochemical studies for sensing applications

Manunya Tepakidareekul,[a] Taro Uematsu,*[a,b] and Susumu Kuwabata*[a,b]

[a]*Department of Applied Chemistry, Graduate School of Engineering, Osaka University, 2-1 Yamada-oka, Suita 565-0871, Japan.*

[b]*Innovative Catalysis Science Division, Institute for Open and Transdisciplinary Research Initiatives (ICS-OTRI), Osaka University, Suita 565-0871, Japan.*

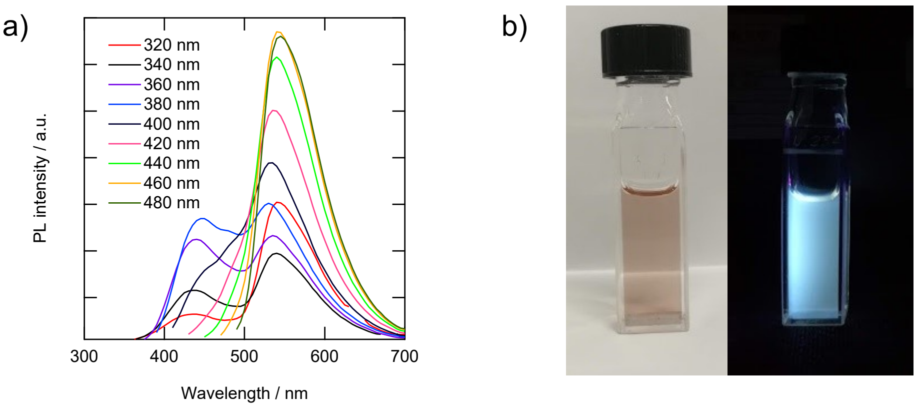

Figure S1.. Photoluminescence spectra (a) of as-prepared CDs measured in aqueous solution at different excitation wavelengths (pH 7.0) and CD solution as prepared in the presence of ambient light and 365 nm UV light (b) .

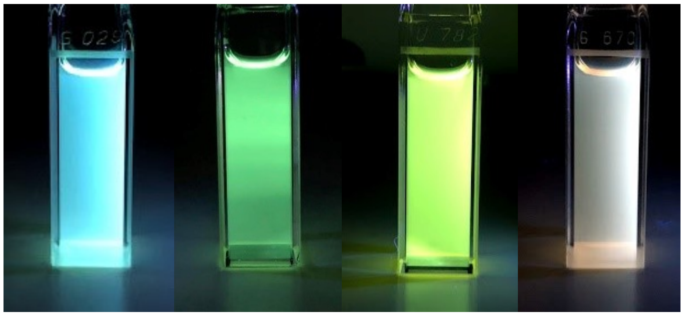

Figure S2. The solutions of the b-CDs, g-CDs, y-CDs, and o-CDs under 365 nm UV irradiation.

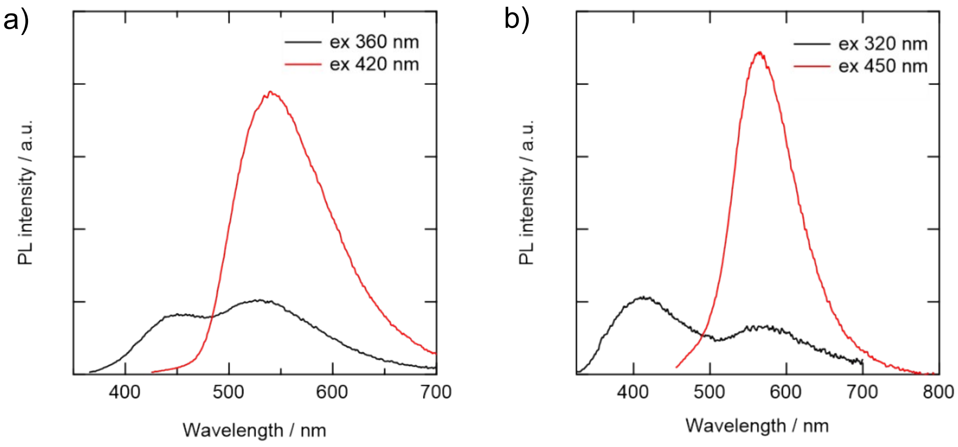

Figure S3. Photoluminescence spectra of the g-CDs (a) and y-CDs (b) were recorded with varied excitation wavelengths in an aqueous solution (pH 7.0).

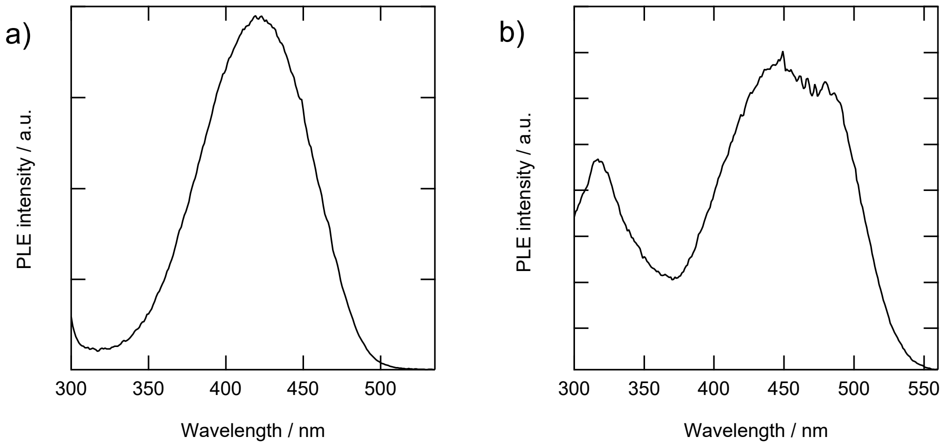

Figure S4. Photoluminescence excitation spectra of the g-CDs (a) and y-CDs (b) were recorded at PL peak wavelengths in an aqueous solution (pH 7.0, 420 nm for g-CDs and 450 nm for y-CDs).

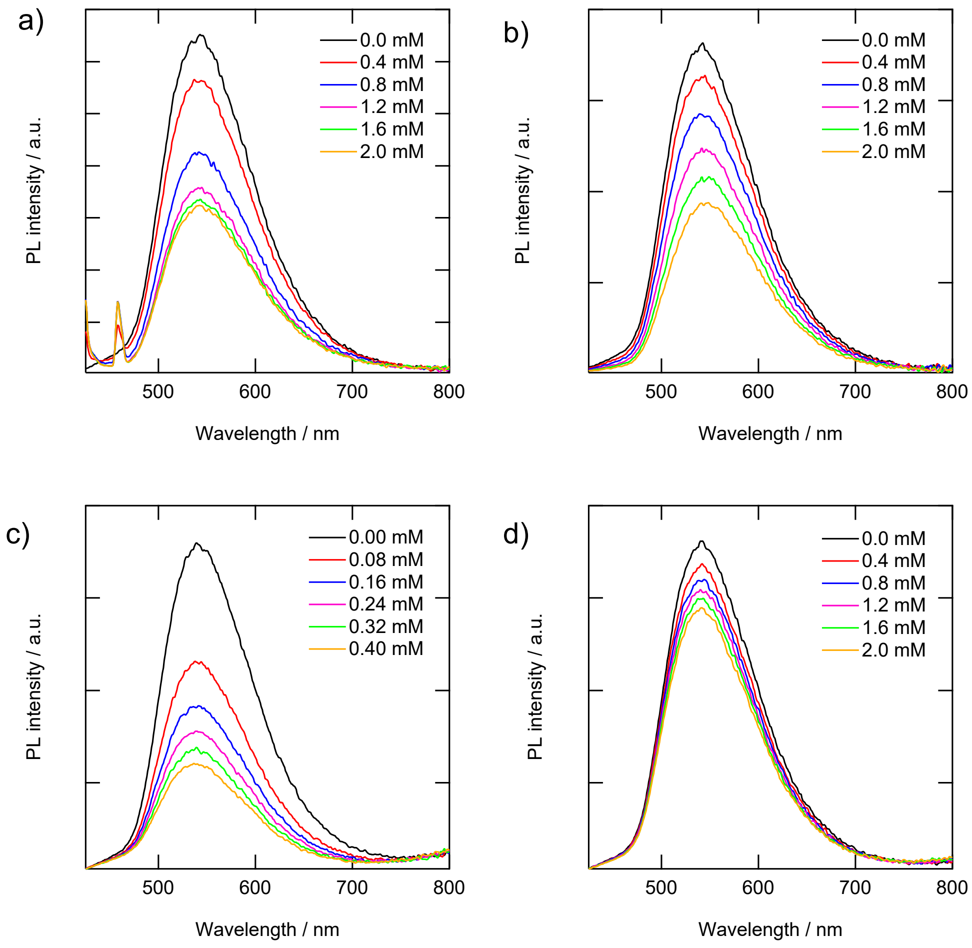

Figure S5. Variations in photoluminescence spectra for the g-CDs by the addition of Ag^{+} (a), Fe^{3+} (b), Cu^{2+} (c), and Co^{2+} (d) in aqueous solution (pH 7.0).

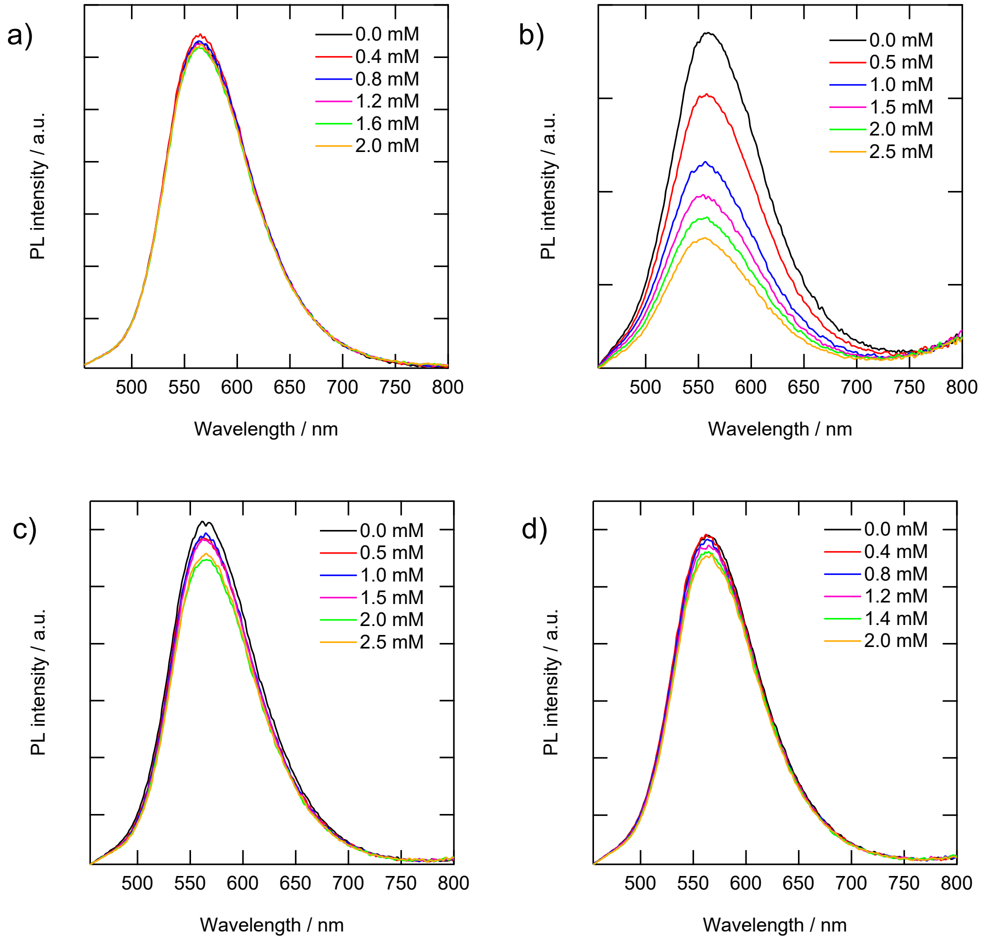

Figure S6. Variations in photoluminescence spectra for the y-CDs due to the addition of Ag^{+} (a), Fe^{3+} (b), Cu^{2+} (c), and Co^{2+} (d) in aqueous solution (pH 7.0).

Table S1 Optical properties of g-CDs and y-CDs.

CDs	λ_{ex} (nm)	λ_{em} (nm)	Photoluminescence quantum yield (%)			
			CH_2Cl_2	CH_3CN	MeOH	H_2O
**g-CDs**	420	540	3	58	36	17
**y-CDs**	450	565	6	20	15	5

Limit of detection calculations

The limit of detection (LOD) is determined from fluorescence titration data based on the literature. The PL spectra of the g-CDs and y-CDs were measured 10 times and the standard deviations of these blank measurements (converted to Stern–Volmer type) were obtained. The detection limit was calculated with the following equation:

$$LOD = 3\sigma/K_S$$

where σ is the standard deviation of the blank measurement and K_S is the slope between fluorescence intensity versus quencher concentration (Figure 6c and 6d in main text). The K_S values were found to be 6645 M^{-1} and 917 M^{-1} for g-CDs and y-CDs, respectively. Therefore, the detection limit of g-CDs toward Cu^{2+} was calculated to be 10.3 µM (LOD = 3(0.0229)/6645 = 1.03×10^{-5} M). For y-CDs towards Fe^{3+}, the detection limit was calculated to be 0.11 mM (LOD = 3(0.0346)/917 = 1.13×10^{-4} M).
